# Reprogramming in delipidated adipocytes: metabolism, regulation and progress

**DOI:** 10.1038/s41420-026-03199-4

**Published:** 2026-06-28

**Authors:** Zhanshuo Chang, Yunjun Liao, Wenqing Jiang

**Affiliations:** Department of Plastic and Cosmetic Surgery, Nanfang Hospital, Southern Medical University, Guangzhou, Guangdong China

**Keywords:** Transdifferentiation

## Abstract

Through specific induction protocols in vitro or under physiological or pathological conditions in vivo, mature adipocytes can be converted into mesenchymal stem cells, challenging our traditional understanding of mature adipocyte identity and cell fate determination. Given that dedifferentiated adipocytes are a rich source of stem cells in the human body, they hold great potential for applications in regenerative medicine. This review discusses the process of lipid droplet depletion and the stage-specific metabolic remodeling of intracellular substances—including triglycerides and free fatty acids, energy-related metabolites (ATP, NAD⁺/NADH, acetyl-CoA), as well as the alterations in key proteins, organelle remodeling, and epigenetic modifications. These insights provide a deeper understanding of adipocyte identity and the molecular mechanisms underlying the transition between different adipocyte differentiation stages. The review also highlights advanced techniques in the field, including adipocyte lineage-tracing models and single-cell RNA sequencing, enhancing the accuracy and precision of research while enabling the capture of dynamic changes within cells. These advancements offer a more comprehensive approach to studying dedifferentiated adipocytes.

## Facts

- Dedifferentiation of adipocytes can be induced in vitro by chemical, cytokine, or stress stimuli, and occurs naturally in vivo during physiological processes like lactation and hair follicle cycling.
- Dedifferentiated adipocytes exhibit stem cell-like properties and can re-differentiate or transdifferentiate into other cell types.
- Dedifferentiation of adipocytes is accompanied by increased activity of lipolytic enzymes, such as ATGL, and inhibition of lipolysis can partially prevent dedifferentiation.

## Open Questions

- The molecular mechanisms and signaling pathways governing adipocyte dedifferentiation remain unclear.
- Is adipocyte dedifferentiation induced by lipolysis? Does lipolysis of adipocytes always lead to its dedifferentiation? The association is not entirely known.
- Whether and how lipolytic activity can be precisely regulated to control dedifferentiation remains to be determined.
- How to controllably induce adipocyte dedifferentiation in vivo for regenerative medicine applications is still unknown.
- The long-term safety and functionality of dedifferentiated cells lack systematic evaluation.

## Introduction

With the rapid advancement of stem cell biology in the 21st century, stem cell therapy has emerged as a promising field for both research and clinical applications, offering significant potential for translational medicine [1]. Stem cell-based regenerative therapies have shown significant clinical efficacy in hematological malignancies, burn wounds, and ocular degeneration [2, 3]. Well-stored with somatic multipotent stem cells, adipose tissue has long been a preferred donor site for stem cell therapy with its ease of collection and abundance of stem cells [4]. A large amount of evidence has demonstrated the safety and efficacy of adipose-derived stem cells in clinical application [5, 6].

In adipose tissue, the stromal vascular fraction (SVF) cells account for approximately 10–30%, while adipocytes make up 70–90%. In addition to adipose-derived stem cells (ASCs) from the SVF, recent studies have shown that adipocytes themselves can also serve as a source of adult stem cells [7–9]. Previously, mature adipocytes were considered to be fully differentiated and devoid of proliferative capacity. However, in 1986, Sugihara first applied a ceiling culture method to mature adipocytes [10]. In this approach, freshly isolated mature adipocytes were cultured in a completely filled culture flask, allowing the buoyant fat cells to float and attach to the inner upper surface of the vessel. During continuous culture, typically over 7–10 days, intracellular lipid droplets were progressively depleted, and the originally unilocular, spherical adipocytes gradually lost their adipocyte morphology and transformed into fibroblast-like cells. This process is firstly referred to as adipocyte dedifferentiation and used as a protocol for in vitro induction. During the shift to dedifferentiated fat (DFAT) cells, markers associated with mesenchymal stem cells (MSCs) such as CD44, CD146, CD266, CD325, and CD332, hematopoietic markers (e.g., CD10, CD24, CD232), and myeloid lineage markers (e.g., CD10, CD13, CD40, CD44) are significantly upregulated, while genes related to lipid metabolism, such as adiponectin (ADIPOQ), hormone-sensitive lipase (LIPE), pyruvate dehydrogenase kinase 4 (PDK4), lipoprotein lipase (LPL), fatty acid synthase (FASN), peroxisome proliferator-activated receptor gamma (PPARG), and fatty acid-binding protein 4 (FABP4) are downregulated [11].

There are more and more in vitro induction of fat dedifferentiation schemes, such as Wnt3a, transforming growth factor-β (TGF-β1) [12], insulin signaling pathway inhibitor OSI-906 [13], TNF-α [14], FIZZ1 [15], and oncostatin M (OSM) [16]. Specific stimuli such as hyperosmotic conditions [17] and cold stress [18] have also been used as in vitro induction methods.

Besides the in vitro induced microenvironments, some physiological conditions also lead to adipocytes dedifferentiation. Adipocytes in mouse breast tissue lose lipid droplets during pregnancy, which the lineage tracing result shows them a preadipocyte-like state transformation [19, 20]. And they could restrore lipid droplets when lactation stops, returning back to the differentiated state. Similarly, the shift of adipocytes were found between the regression phase and anagen phase of the hair follicle growth cycle [21].

In some pathological conditions, such as bone tumors like osteosarcoma [22] and chondrosarcomaduring free fat grafting for clinical reconstruction use, some mature adipocytes also change into fibroblast-like cells under ischemic hypoxic conditions [23]. However, whether these cells could be designated as a dedifferentiated state needs a more precise definition and further verification.

Despite the broad prospects of DFAT cells for tissue regeneration and regenerative medicine, research on dedifferentiated adipocytes is still incomplete. Whether for in vitro transplantation or in vivo induction, their safety and efficacy have not yet been fully addressed. DFAT cells do not exclusively confer regenerative benefits and, in certain pathological contexts, may instead promote tumor progression, highlighting its dualistic nature. Within the tumor microenvironment, dedifferentiated adipocytes can participate in extracellular matrix remodeling, inflammatory signaling, and metabolic crosstalk that supports tumor growth, invasion [24]. In conclusion, a key unresolved challenge lies in achieving precise and context-specific regulation of adipocyte dedifferentiation. This includes selectively activating dedifferentiation to support tissue repair and regenerative therapies, while effectively suppressing maladaptive dedifferentiation that contributes to tumor progression or pathological remodeling, ensuring the long-term safety, functionality, and minimizing potential side effects, so that they can be applied in therapies. Given the wide application potential of dedifferentiated adipocytes in regenerative medicine, they will undoubtedly be a hot research topic in the future. Therefore, we aim to clarify how dedifferentiated states can be defined, identify the initiating signals and regulatory cues that drive dedifferentiation, and explore strategies for achieving precise control of this process.

## Reprogrammed progenitor-like features

DFAT cells lack specific cell surface markers, and the mechanisms underlying their dedifferentiation process remain poorly understood, for which current research could only focus on their morphology and surface markers [19, 21, 25].

Dedifferentiation of adipocytes is seen as almost the reversed processes of differentiation. From a commited differentiated state to progenitor phenotype then to owning the ability of multi-directional differentiation, DFAT cells experience not only the phenotypic changes but also nucleus remodeling for increasing proliferative and pluripotent potential, dynamic protein changes adapting activated and quiescent organelles and adjustive metabolic changes with intracellular components rearrangement.

Though recent studies displayed only indistinct changes of DFAT cells during the dedifferentiation process and haven’t defined any their undergoing stages yet, we may draw all these threads together and try relating them to the reversed differentiation states to better understand the mechanisms governing the cellular remodeling and further elucidate the initiating factors and cascading triggers of adipocyte dedifferentiation.

## Phenotypic changes

Current consensus on the determined phenotype of DFAT cells only includes the surface markers and morphology [26]. DFAT cells can be divided into several states according to the surface markers: adipocyte precursor cells or adipocyte progenitor cells, uncommitted adipocyte, committed adipocyte, differentiating adipocyte, mature adipocyte [27]. Hallmark genes of mature adipocytes can be functionally grouped into several categories. FABP4 and PPARγ act as central regulators of fatty acid metabolism and adipocyte-specific transcription. LPL, ADIPOQ, and LEP reflect lipid uptake and endocrine activity. Lipe and Pnpla2 control lipid droplet turnover and hormonally regulated lipolysis. The coordinated downregulation of these genes during adipocyte dedifferentiation indicates a global loss of mature adipocyte metabolic and functional identity. But markers of preadipocytes (PDGFα, PDGFβ) and fibroblasts (VIM, COLLA1) increase. In single-cell RNA sequencing, individual cells are distinguished based on their transcriptional profiles [28]. Cells with similar gene-expression patterns naturally cluster together, and by integrating known cell-type markers, these clusters can be classified into distinct cellular subpopulations. By combining genetic lineage tracing with single-cell RNA sequencing, mature adipocyte–derived cells can be unambiguously identified and their fate tracked at single-cell resolution, enabling reconstruction of cell state transition trajectories during dynamic processes [29, 30].

Mature adipocytes exhibit a characteristic morphology dominated by a single, large unilocular lipid droplet that occupies nearly the entire cell volume. The cells are typically round in shape, with a large diameter, while the nucleus and cytoplasm are compressed to the cell periphery. During dedifferentiation, adipocytes undergo pronounced morphological remodeling [31]. The unilocular lipid droplet gradually breaks down into multiple smaller lipid droplets, resulting in a multilocular appearance. Concomitantly, the overall cell size decreases, although cells largely maintain a rounded morphology at this stage. After dedifferentiation is completed, adipocyte-derived cells lose detectable lipid droplets and display an elongated, spindle-shaped morphology resembling fibroblast-like cells. These cells exhibit a flattened appearance and reduced diameter, consistent with the morphology of adipocyte progenitors or other low-differentiation mesenchymal cell types [32].

The morphological features of adipocyte dedifferentiation closely resemble the cellular changes observed during adipose tissue atrophy in vivo. Under conditions of adipose tissue shrinkage, mature adipocytes commonly display reduced lipid droplet content, decreased cell size, and altered cellular morphology. The morphological features observed during adipocyte dedifferentiation closely resemble the cellular changes that occur during adipose tissue atrophy in vivo. During adipose tissue shrinkage, mature adipocytes typically exhibit reduced lipid droplet content, decreased cell size, and marked alterations in cellular morphology. Consistent with these observations, similar phenotypes have been reported in multiple genetic models. For instance, adipocyte-specific deletion of Lats1/2 in mice leads to pronounced adipose tissue atrophy, accompanied by loss of mature adipocyte differentiation programs and the emergence of dedifferentiation phenotypes [33]. Comparable features have also been observed in Ube2i knockout mice [34]. These findings once again suggest that changes in adipocyte phenotype and gene expression are closely linked to adipose tissue reduction.

## Nucleus remodeling and proliferative activity

The nucleus serves as the repository of genetic information and undergoes significant remodeling during cell differentiation. As preadipocytes mature into adipocytes, the nucleus decreases in size and is progressively displaced from the center to the periphery due to the expansion of lipid droplets. This results in an irregular, flattened, or crescent-like nuclear morphology.

Verstraeten et al. demonstrated that during adipogenesis in 3T3-L1 pre-adipocytes, nuclear lamina reorganization drives changes in nuclear shape and positioning [35]. The inner nuclear membrane is composed of A-type and B-type lamins, which form the nuclear lamina and LINC (Linker of Nucleoskeleton and Cytoskeleton) complex [35]. During adipogenesis, A-type lamins migrate from the nuclear interior to the periphery, accompanied by an increase in abundance and increase in abundance, softening the nucleus and enhancing its deformability. This restructuring facilitates the irregular nuclear positioning observed in mature adipocytes [36]. Additionally, structural changes in Lamin A influence chromosomal positioning, leading to gene activation and transcriptional regulation [37]. These nuclear reorganizations are critical for coordinating adipocyte gene expression and function.

Lipodystrophy, a disease characterized by generalized or localized fat atrophy and metabolic disorders such as insulin resistance, fatty liver, and hyperlipidemia, is commonly linked to mutations in the LMNA gene that encodes lamin A and C [38]. These laminopathies disrupt nuclear membrane integrity, which is crucial for cell migration and remodeling. During adipocyte dedifferentiation, the nucleus undergoes dynamic structural modifications to support functional transitions. Various physical stimulation, including increased extracellular matrix stiffness, mechanical compression, and osmotic stress, can alter cellular and nuclear mechanical tension [17]. Through LMNA dependent regulation of nuclear lamina tension [39], these mechanical inputs promote the nuclear localization of the mechanosensitive transcriptional coactivator YAP [40], thereby driving downstream transcriptional reprogramming. Nuclear YAP and TAZ interact with the TEAD transcription factor family to regulate the expression of specific genes, thereby affecting cell proliferation, migration, differentiation, and cell fate determination. Recently, Lei established an adipocyte–tumor co-culture assembloid system in which nuclear morphology of both adipocytes and cancer cells was systematically analyzed. Internal mechanical stresses within the assembloids were quantified using the jelly–pearl mathematical model, revealing that stress redistribution during assembloid fusion was reflected by pronounced changes in nuclear area. Following culture, adipocytes located in the junctional zone exhibited reduced nuclear areas, concomitant with enhanced adipocyte dedifferentiation and increased tumor cell proliferative capacity in this region [41]. These findings suggest that mechanically generated internal stresses within assembloids may induce nuclear deformation, thereby promoting adipocyte state plasticity and cell fate remodeling.

## Multipotent features acquisition

In dedifferentiated adipocytes, genes related to migration, proliferation, tissue development, and morphology are significantly upregulated, suggesting that DFATs display features of tissue-specific progenitors [11]. Similar to ASCs, DFAT cells hold promise for regenerative medicine. DFATs show regenerative capacity across multiple tissues. They can differentiate into osteoblasts under retinoic acid or BMP-2 induction [42]. In myocardial infarction models, DFAT cells transplantation promotes myofibril formation and angiogenesis [43]. They contribute to smooth and skeletal muscle regeneration in the urinary system and provide renal protection in vesicoureteral reflux models [9]. Beyond direct differentiation, DFAT cells also remodel the microenvironment by secreting cytokines and chemokines, thereby recruiting mesenchymal and endothelial cells to support tissue repair [44–46].

## Dynamic protein changes

Biological processes depend on the precise spatial organization and dynamic remodeling of intracellular protein networks. Understanding protein dynamics—both in abundance and subcellular localization—is essential for elucidating cellular functions. In adipocytes, these protein changes are closely linked to lipid droplet formation, lipid metabolism, signaling, and cytoskeletal remodeling, all of which are critical in determining adipocyte fate. The transition from fibroblast-like precursors to rounded, lipid-laden adipocytes relies on dynamic cytoskeletal remodeling, and disruption of this process impairs differentiation. During dedifferentiation, cytoskeletal reorganization and signaling pathways such as Wnt/β-catenin, TGF-β, Notch, and Hippo also play key roles [26].

## Wnt/β-catenin signaling pathway

In mature adipocytes, canonical Wnt signaling is typically repressed; however, it becomes reactivated during adipose tissue remodeling and adipocyte dedifferentiation. Upon Wnt stimulation, β-catenin escapes degradation by the AXIN–APC–GSK3β–CK1 destruction complex, accumulates in the cytoplasm, and translocates into the nucleus [47]. Nuclear β-catenin associates with TCF/LEF transcription factors, BCL9/BCL9L docking proteins, and PYGO coactivators to induce transcription of genes that directly antagonize adipocyte fate, including C-MYC, CCND1, PPARδ, and COUP-TFII [48, 49]. These targets suppress adipocyte identity through multiple mechanisms: C-MYC inhibits C/EBPα expression; CCND1 and PPARδ inhibit PPARγ transcriptional activity; and COUP-TFII recruits the SMRT corepressor complex to intronic regulatory regions of PPARγ1 and PPARγ2, leading to local histone hypoacetylation and transcriptional silencing of PPARγ. Collectively, these mechanisms destabilize the adipogenic transcriptional network and facilitate reversion to a low-lipid, low-PPARγ state [50].

## TGF-β/SMAD3 signaling pathway

Binding of TGF-β1 to TβRII results in activation of TβRI and subsequent phosphorylation of SMAD2 and SMAD3. Phosphorylated SMAD2/3 form complexes with SMAD4 and translocate into the nucleus, where they suppress the transcriptional activity of early adipogenic regulators C/EBPβ and C/EBPδ [51, 52]. Because these factors are essential for sustaining PPARγ expression and adipocyte phenotype stability, their inhibition leads to reduced PPARγ mRNA levels, impaired lipid synthesis, and loss of adipocyte-specific gene expression. In experimental models, enhanced SMAD3 signaling closely correlates with lipid droplet depletion and acquisition of fibroblast-like morphology [51].

## Notch signaling pathway

**Fig. 1:**
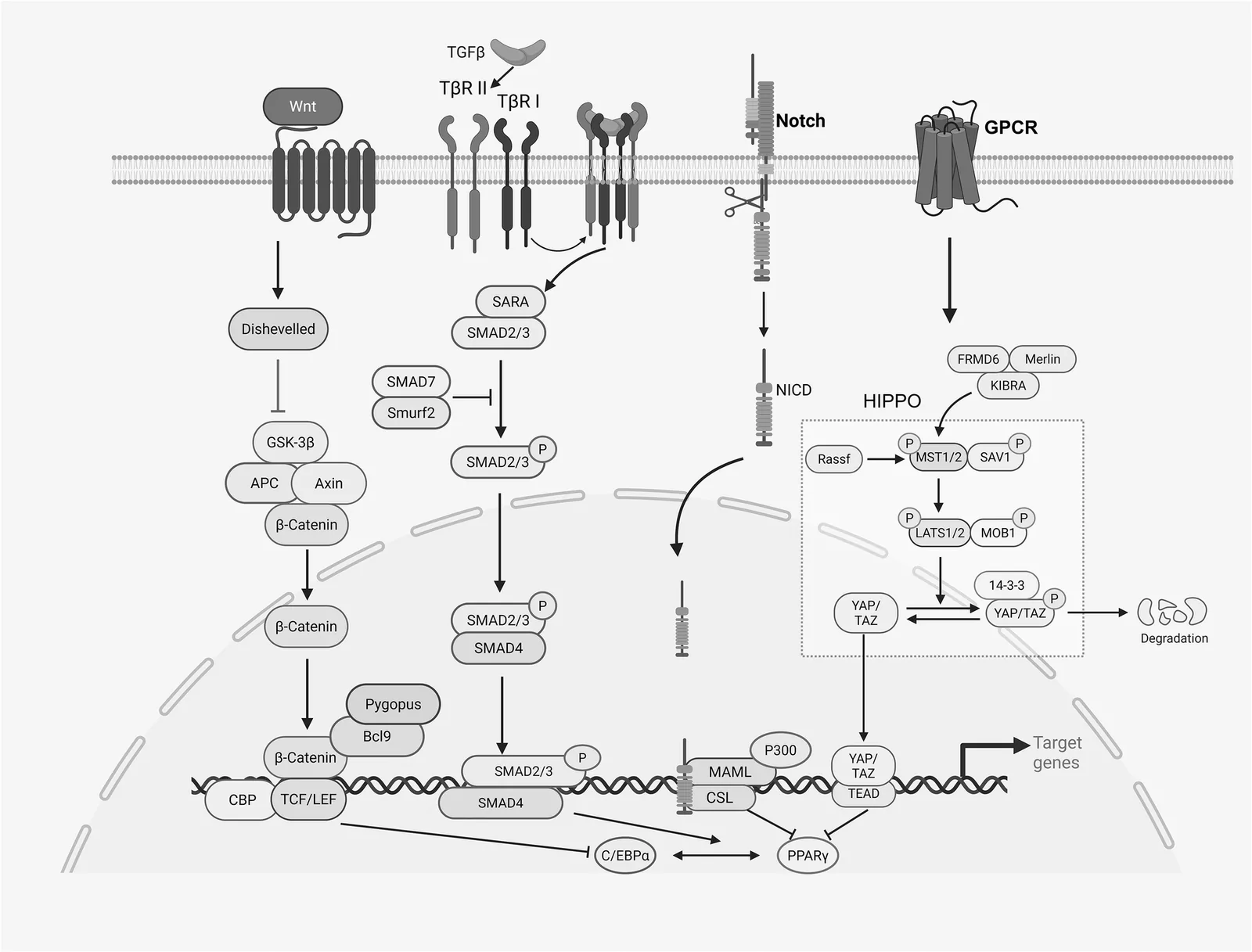
Regulation of cell fate and phenotypic plasticity by TGF-β, Notch, Wnt, and Hippo signaling pathways. TGF-β signaling is initiated by ligand binding to TGF-β receptors, leading to SMAD2/3 phosphorylation and nuclear translocation to regulate target gene transcription. Notch signaling is activated through ligand–receptor interaction between adjacent cells, resulting in the release of the Notch intracellular domain (NICD), which translocates into the nucleus and cooperates with transcriptional regulators. Canonical Wnt signaling stabilizes β-catenin by inhibiting its degradation complex, allowing β-catenin to accumulate and enter the nucleus to activate Wnt target genes. Hippo signaling controls the phosphorylation status of YAP/TAZ; when Hippo signaling is inactive, YAP/TAZ translocate into the nucleus and function as transcriptional co-activators. Extensive crosstalk exists among these pathways at multiple levels, including shared transcriptional regulators, cooperative or antagonistic interactions in the nucleus, and coordinated regulation of cell fate decisions, proliferation, differentiation, and tissue remodeling.

The Notch signaling pathway promotes adipocyte dedifferentiation by reinforcing progenitor-like transcriptional programs. Ligand-induced activation of Notch receptors leads to cleavage and release of the Notch intracellular domain (NICD) [53], which translocates to the nucleus and interacts with RBPJ to activate downstream targets such as HES-1, HEY-1, and PREF-1. PREF-1, through activation of the MEK/ERK pathway, induces SOX9, which directly binds to the promoters of C/EBPβ and C/EBPδ, suppressing their transcription and thereby preventing maintenance of the adipocyte differentiation program [54]. In parallel, HES-1 directly represses PPARγ transcription by targeting E-box elements within PPARγ promoters. Through this dual inhibitory mechanism, Notch signaling simultaneously blocks adipogenic transcriptional cascades and stabilizes a less differentiated cellular state [55]. The above signaling pathways are summarized in Fig. 1.

Detailed protein changes and references are summarized in the Table 1.

**Table 1:** Dynamic changes of different protein types in adipocyte differentiation.

Feature	Proteintype	Specific protein examples	Functions
Early down	Actin/cytoskeleton	F-actin, Tubulin, Actin, Filamin, Coactosin, NEDD5	Maintain cell morphology, motility, division, and molecular transport; decrease during differentiation, except coactosin is upregulated [121]; NEDD5 decreases after 48 h, associated with growth arrest [122, 123]
	Cell motility	FSCN1/CNN2	Participate in processes such as cell growth, division, and migration
	Cell cycle	Cyclin-dependent kinase 1/PCNA	Ensure smooth progression of cell division; dedifferentiation requires re-entry into the cell cycle [45, 124]
Middle up	TCA metabolism	Pyruvate carboxylase/Isocitrate dehydrogenase	Oxidize organic compounds efficiently to meet metabolic demands [125]
	Oxidative phosphorylation	Electron carrier enzymes/ATP synthases	Generate energy and maintain metabolic homeostasis [125]
	Triglyceride metabolism	α-Ketoglutarate dehydrogenase/NAMPT	Contribute to lipid breakdown and energy generation [125]
Late up	Perilipins family	Perilipin1/Perilipin2	Regulate dynamic lipid droplet remodeling through interaction with lipids on their surface
	Fatty acid metabolism	Fatty Acid Synthase/Adipose Triglyceride Lipase	Participate in fatty acid synthesis, β-oxidation, transport, and regulation
Mechanistic links	ECM and cytoskeleton	Actin filaments, Microtubules	ECM mechanical compression induces dedifferentiation, requiring actin and microtubule reorganization to transition cells from lipid-filled spheres to fibroblast-like forms [126]
	Signaling pathways	Rho GTPases, PI3K/Akt	Cytoskeletal dynamics modulate signaling cascades that influence proliferation, polarity, and dedifferentiation [127, 128]

The table summarizes major categories of proteins, representative examples, and their functions at different stages of dedifferentiation. Early downregulated proteins involve cytoskeleton and cell cycle regulation, mid-phase upregulation supports metabolism and energy production, and late upregulated proteins control lipid droplet remodeling. ECM–cytoskeleton interactions and key signaling pathways drive the dedifferentiation process.

## Cellular metabolic changes

### Lipid depletion—from adipocytes to DFAT cells

A key feature of adipocyte dedifferentiation is the progressive loss of lipid droplets. As adipocytes transition to DFAT cells, they lose their rounded shape, lipid droplets diminish, and the cytoplasm regains a fibrous appearance [10]. While lipid droplet secretion plays a crucial role in this process, the underlying mechanisms remain unclear.

Kim et al. reported a ceiling culture chip integrated with live-cell imaging that enables long-term visualization of adipocyte dedifferentiation under stable ceiling culture conditions [56]. Using this system, adipocytes underwent pronounced morphological remodeling accompanied by dynamic lipid droplet (LD) extrusion. During this process, prominent pseudopodia formed at the apical surface, and actin filaments were redistributed around LDs to generate an actin “shell.” In some cells, pores of varying sizes emerged within this actin shell and subsequently fused into one or two larger openings that functioned as extrusion channels. Actin contraction then drove the expulsion of plasma membrane–encapsulated LDs, leaving behind characteristic hollow actin ring structures. These observations illustrate a distinct mode of delipidation during adipocyte dedifferentiation, namely LD secretion [56].

In the tumor microenvironment, cancer-associated adipocytes (CAAs) exhibit phenotypic features highly consistent with adipocyte dedifferentiation. Compared with mature adipocytes, CAAs display marked loss of LDs and downregulation of adipocyte differentiation markers, including ADIPOQ, PPARγ, and PLIN1, while acquiring fibroblast-like morphology and mesenchymal and inflammatory gene expression signatures. This phenotypic transition is accompanied by enhanced lipolytic activity and sustained release of free fatty acids [57]. Consistently, genetic ablation of ATGL in bone marrow stromal cells impairs adipocyte dedifferentiation [58]. Moreover, lineage-tracing studies of mature adipocytes in mouse wound models, combined with single-cell RNA sequencing and transcriptomic analyses, demonstrate that lipolysis can induce adipocyte dedifferentiation and give rise to heterogeneous myofibroblast populations that contribute to extracellular matrix deposition in the wound bed [59]. Together, these findings indicate that lipolysis represents another major mechanism of delipidation driving adipocyte dedifferentiation.

The emergence of these two distinct delipidation modes—lipid droplet extrusion and lipolysis-driven lipid depletion—appears to be largely determined by the surrounding microenvironment and the nature of external cues imposed on adipocytes. Under ceiling culture conditions, adipocytes experience sustained physical confinement and mechanical tension. The upward orientation and external mechanical pulling generate cytoskeletal stress, which is sensed and transduced through the actin network. This mechanical stimulation promotes actin reorganization and contractility, ultimately driving the formation of actin shells and the physical extrusion of lipid droplets [60, 61]. In this context, delipidation is primarily governed by biomechanical forces and cytoskeleton-mediated membrane remodeling.

Differently, within the tumor microenvironment, adipocyte delipidation is predominantly regulated by biochemical signaling rather than direct mechanical stress. Cancer cells establish close contact with cancer-associated adipocytes through gap junctions, enabling the intercellular transfer of second messengers such as cyclic AMP (cAMP) [62]. Elevated intracellular cAMP levels activate protein kinase A (PKA) signaling, leading to phosphorylation and activation of key lipolytic enzymes, including ATGL and HSL [63]. This sustained lipolytic state promotes progressive lipid droplet breakdown, free fatty acid release, and metabolic reprogramming, thereby driving adipocytes toward a dedifferentiated, fibroblast-like phenotype [17]. Together, these observations suggest that adipocyte delipidation during dedifferentiation is a context-dependent process, in which mechanical cues favor lipid droplet extrusion, whereas tumor-associated paracrine and juxtacrine signaling preferentially induce lipolysis-mediated lipid loss.

### Lipophagy

In addition to lipolysis, lipophagy represents another important mechanism for lipid removal in adipocytes. Unlike lipolysis, which enzymatically hydrolyzes triglycerides stored in LDs, lipophagy selectively delivers LDs to lysosomes, where lysosomal acid lipase (LAL) mediates lipid degradation. This process involves LC3-dependent recognition of LD-associated autophagy receptors, interaction with ATGL, and subsequent autophagosome–lysosome fusion [64].

Although lipophagy has been extensively studied in hepatocytes, its role in adipocytes and adipocyte dedifferentiation remains incompletely defined. Available evidence suggests that lipophagy and lipolysis act in a coordinated manner, targeting distinct LDs rather than functioning redundantly. For example, ATGL deficiency leads to the accumulation of large LDs, whereas lysosomal inhibition preferentially increases small LDs, indicating size-dependent lipid clearance pathways. Importantly, lipophagy appears to facilitate lipid turnover and homeostasis during adipocyte remodeling, but lipid removal through lipophagy alone does not necessarily trigger dedifferentiation [65].

### Lipase-independent lipid release

Beyond enzymatic lipid degradation, adipocytes can also mobilize lipids through a lipase-independent pathway involving the release of exosome-like vesicles derived from lipid droplets [66]. In this process, triglycerides are exported without prior hydrolysis, encapsulated within membrane-bound vesicles that originate from the intracellular membrane system. Although this mechanism accounts for a relatively small fraction of total lipid efflux, it represents a distinct mode of delipidation.

Adipocyte-derived extracellular vesicles have been shown to transfer triglycerides to neighboring cells, such as macrophages, supporting intercellular metabolic communication within adipose tissue. However, whether vesicle-mediated lipid release directly contributes to adipocyte dedifferentiation or primarily serves as a mechanism for lipid redistribution remains unclear. Current evidence suggests that this pathway facilitates lipid clearance without necessarily inducing a full loss of adipocyte identity.

**Fig. 2:**
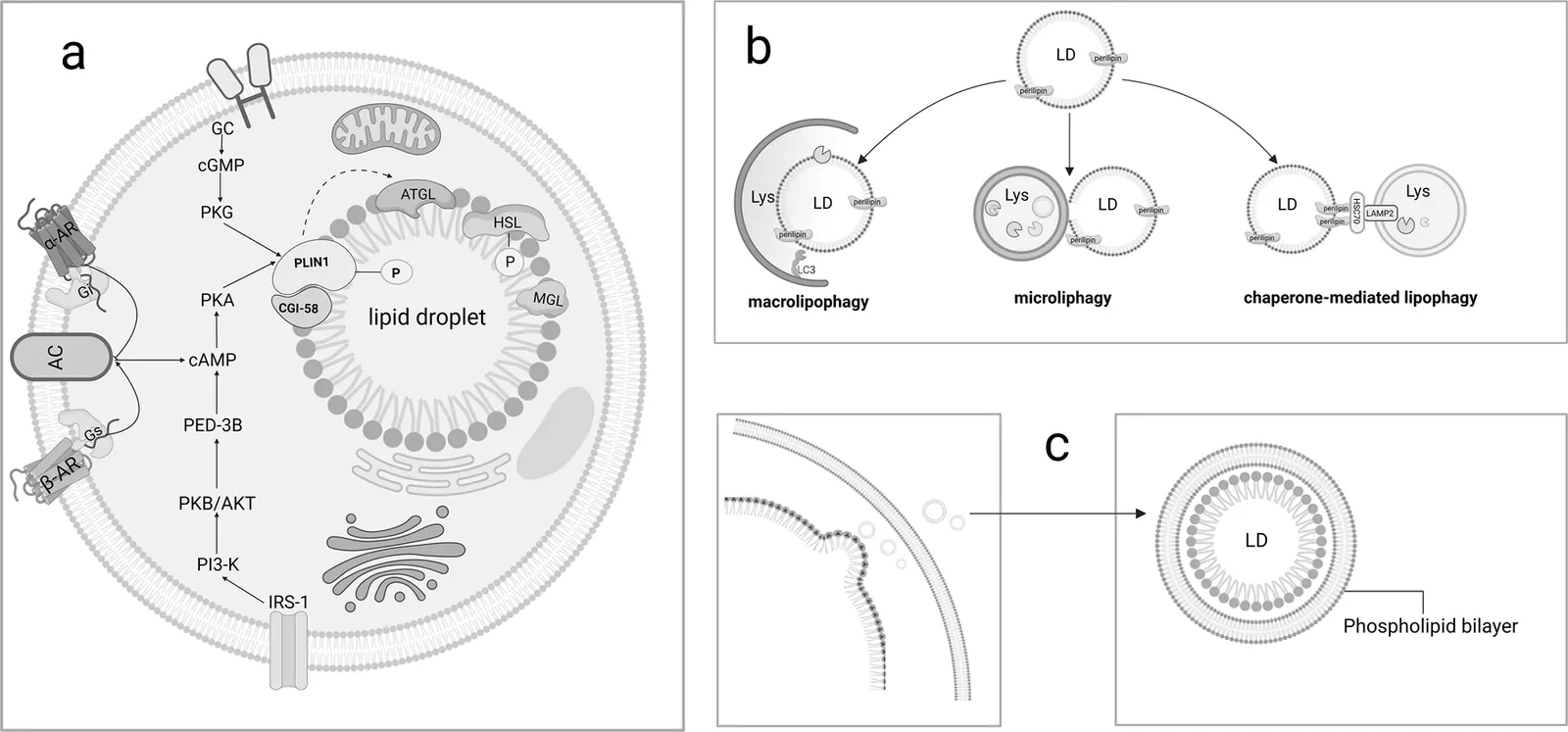
Pathways of lipid mobilization in adipocytes. **a** Classical lipolysis–Hormone-sensitive signaling (cAMP/PKA, cGMP/PKG) regulates phosphorylation of PLIN1 and HSL, releasing CGI-58 to activate ATGL for stepwise hydrolysis of triglycerides (TG → DG → MG → FA + glycerol). Lipolysis is stimulated by catecholamines and natriuretic peptides, while insulin and adenosine suppress it via PI3K-AKT–PDE3B–mediated cAMP degradation. **b** Lipophagy–Lipid droplets are degraded via autophagic pathways, including macroautophagy (LC3-II–dependent autophagosome–lysosome fusion), microautophagy (RAB-mediated lysosomal engulfment), and chaperone-mediated autophagy (CMA) targeting droplet-coating proteins (HSP70/LAMP2), thereby facilitating lipase access. **c** Lipase-independent lipid release–Adipocytes secrete triglyceride-rich lipid droplets as exosome-like vesicles surrounded by a phospholipid bilayer containing droplet proteins (e.g., PLINs, ATGL, CD63), which can be transferred to recipient cells such as macrophages.

These findings indicate that adipocytes employ multiple mechanisms to eliminate lipid stores, including lipolysis, lipophagy, and vesicle-mediated lipid export, as summarized in Fig. 2. While adipocyte dedifferentiation is invariably accompanied by lipid loss, delipidation itself represents a complex and context-dependent process. Current evidence remains insufficient to determine whether adipocyte dedifferentiation is driven by one dominant delipidation pathway or by the coordinated action of several lipid-removal mechanisms operating in parallel or sequentially. Moreover, it remains unclear whether specific modes of delipidation instruct cell fate transitions or merely facilitate structural changing during dedifferentiation. Elucidating how these distinct lipid-handling processes are integrated, and under which conditions they converge to promote dedifferentiation, remains an important unresolved question in the field.

### Changes in lipid metabolism

A hallmark of adipocyte differentiation is the progressive accumulation of lipids, whereas reduced differentiation or dedifferentiation is primarily characterized by a decrease in lipid droplet number and size. The most prominent metabolic change during differentiation is the rapid synthesis and stable storage of triglycerides (TAGs) [67]. Concomitantly, adipocyte differentiation is accompanied by a systematic remodeling of fatty acid composition: the relative abundance of polyunsaturated fatty acids and very-long-chain fatty acids markedly declines in mature adipocytes, whereas short-chain and monounsaturated fatty acids (MUFAs) become progressively enriched. Oates et al. quantitatively analyzed fatty acid accumulation during adipocyte differentiation and found that C18:1 and C16:0 predominate in preadipocytes and early differentiation stages, whereas C16:1, C16:0, and C15:0 become significantly enriched in mature adipocytes, with a pronounced increase in the proportion of monounsaturated fatty acids as differentiation progresses [68]. Short-chain and monounsaturated fatty acids are more efficiently utilized for triglyceride synthesis and are more readily incorporated into stable neutral lipids, thereby meeting the high demand for large-scale lipid storage in adipocytes [69]. In addition, an increased proportion of short fatty acids enhances the fluidity and flexibility of cellular and lipid droplet membranes, facilitating adipocyte adaptation to dynamic changes in cell volume and functional requirements [70]. Moreover, short-chain unsaturated fatty acids exhibit higher metabolic efficiency during β-oxidation, enabling rapid energy production and supporting metabolic flexibility as well as antioxidant defense in adipocytes [71]. These systematic changes in fatty acid composition reflect the establishment of a lipid storage–oriented metabolic program during adipocyte differentiation.

In contrast to the “lipid storage–oriented” metabolic state established during differentiation, adipocyte dedifferentiation is characterized by pronounced metabolic reprogramming of lipid metabolism. Dedifferentiation is accompanied by activation of lipolytic pathways, leading to triglyceride breakdown and fatty acid release. Studies have shown that skin injury induces lipolytic responses in dermal adipocytes, resulting in the selective release of saturated fatty acids (SFAs) and monounsaturated fatty acids (MUFAs) into the wound site in parallel with adipocyte dedifferentiation [59]. Similarly, within the tumor microenvironment, dedifferentiated adipocytes exhibit marked lipid metabolic reprogramming, with particularly prominent alterations in monounsaturated fatty acids, such as palmitoleic acid (C16:1) and oleic acid (C18:1) [72]. Together, these findings demonstrate that adipocytes establish and switch between distinct lipid metabolic programs during differentiation and dedifferentiation, highlighting their remarkable metabolic plasticity. The released fatty acids were not random or uniform, but showed the characteristics of selective mobilization, especially enriched in monounsaturated fatty acids and polyunsaturated fatty acids, which have signaling functions. Recent studies have further demonstrated that CCAs undergo a metabolic reprogramming driven by the Zeb1–Scd axis. This process selectively regulates the synthesis and release of specific monounsaturated fatty acids, particularly palmitoleic acid and oleic acid. These MUFAs act as key paracrine metabolic signals, significantly promoting the proliferation, invasion, and migration of tumor cells. By blocking the Scd-mediated generation of MUFAs through genetic or pharmacological means, the pro-tumor effects of CCAs are effectively eliminated [72].

### Changes in fatty acid precursors during adipocyte differentiation

During adipocyte differentiation, both fatty acid composition and the supply of metabolic substrates undergo profound changes. Overall, differentiation is accompanied by increased lactate secretion, enhanced catabolism of branched-chain amino acids (BCAAs), and a metabolic shift of glutamine from sustained consumption toward relative accumulation [68]. These metabolic features reflect the dynamic adaptation of adipocytes to stage-specific energy demands and pathway selection during differentiation.

**Fig. 3:**
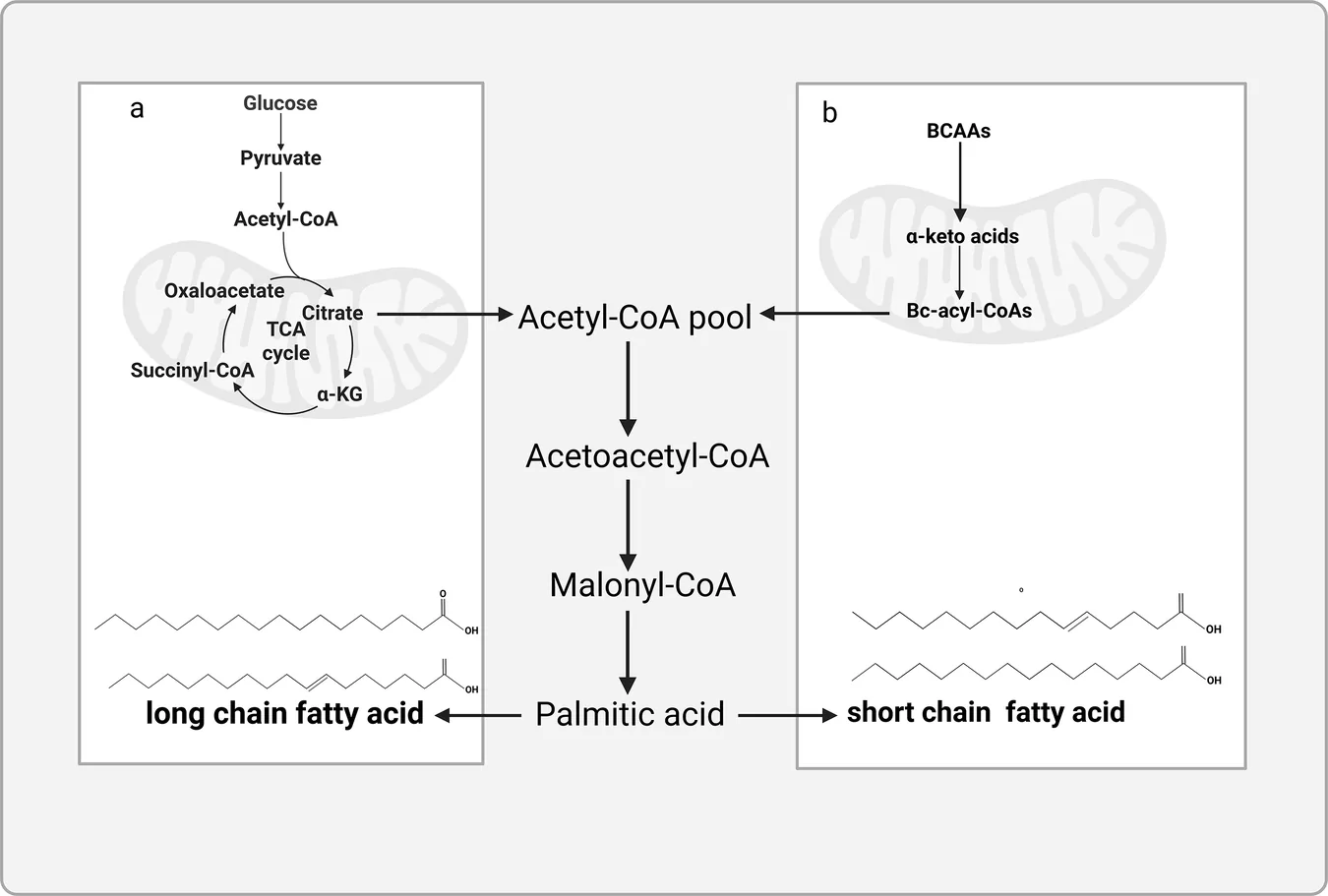
Metabolic shifts in fatty acid precursor utilization during adipocyte differentiation. **a** In preadipocytes and early differentiation stages, glucose and alanine serve as the main sources of acetyl-CoA, and C18:1 and C16:0 are the predominant fatty acids. **b** In mature adipocytes, branched-chain amino acids become the major acetyl-CoA source, with a shift in fatty acid composition toward enrichment of C16:1, C16:0, and C15:0, notably with increased C16:1.

At the undifferentiated preadipocyte stage, cells are in a highly proliferative state with elevated requirements for protein and nucleotide synthesis. During this phase, glutamine serves as a critical nitrogen donor, providing essential substrates for the synthesis of purines, pyrimidines, and multiple non-essential amino acids, thereby supporting cell proliferation and anabolic biosynthesis [73]. As differentiation progresses, adipocytes gradually transition from a growth-oriented metabolic program to a storage-oriented metabolic state, characterized by a markedly increased demand for acetyl–coenzyme A (acetyl-CoA), the key precursor for fatty acid synthesis [74]. Metallo et al. demonstrated that the sources of acetyl-CoA differ substantially across differentiation stages: in preadipocytes, acetyl-CoA is primarily derived from glucose and alanine metabolism, whereas in mature adipocytes it relies more heavily on BCAA catabolism [75]. This substrate switch provides a metabolic explanation for the reduced glutamine utilization and enhanced BCAA breakdown observed during adipocyte differentiation. These stage-specific metabolic transitions during adipocyte differentiation are schematically summarized in Fig. 3.

In sharp contrast, during breast cancer microenvironment–induced dedifferentiation, adipocytes no longer maintain a lipogenesis-dominant metabolic program but instead undergo pronounced metabolic reprogramming toward a stress-adaptive state focused on rapid energy acquisition and environmental adaptation. Dedifferentiated adipocytes exhibit a markedly enhanced glycolytic phenotype; glycolytic stress assays reveal significantly higher basal and compensatory glycolytic rates in dedifferentiated adipocytes compared with normal adipocytes, indicating a strong dependence on glycolytic pathways [57]. This shift reflects a rapid energy-producing strategy adopted by dedifferentiated adipocytes to meet increased energetic and biosynthetic demands under tumor-associated metabolic stress.

Further metabolomic analyses reveal that dedifferentiated adipocytes display pronounced nutrient-dependent metabolic plasticity. Under nutrient-replete conditions, dedifferentiated cells are predominantly enriched in aminoacyl-tRNA biosynthesis and pyrimidine metabolism pathways, suggesting elevated translational capacity and active nucleotide turnover that may support cellular remodeling and phenotype maintenance [57]. In contrast, under nutrient-limited conditions, these cells preferentially activate alanine, aspartate, and glutamate metabolism, as well as glycolysis/gluconeogenesis pathways, thereby sustaining energy production and metabolic homeostasis through amino acid catabolism and carbon flux reprogramming.

### Mitochondria

**Fig. 4:**
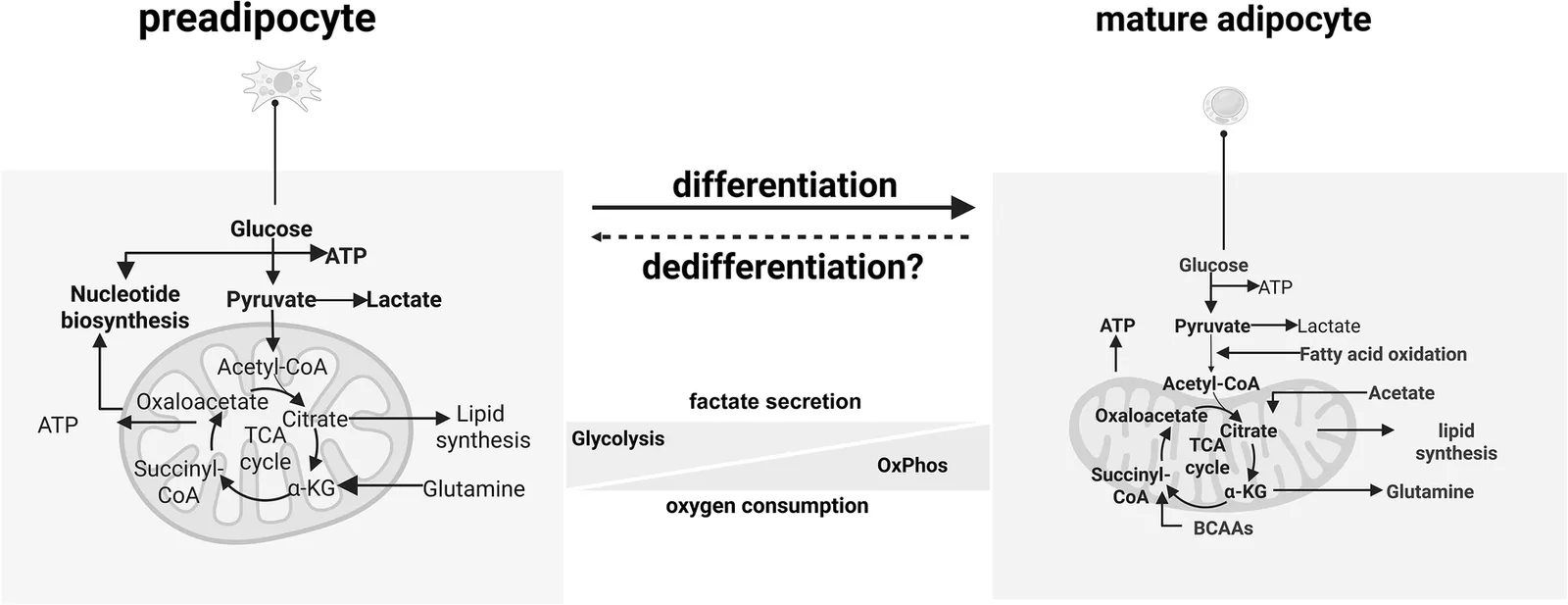
Schematic illustration of mitochondrial remodeling and metabolic reprogramming during adipocyte differentiation. During adipocyte differentiation, mitochondria remodel from small, spherical, perinuclear structures with poorly developed cristae into elongated, tubular, and branched organelles with well-defined cristae. Concomitantly, energy metabolism shifts from a glycolysis-dominant state in early stages to oxidative phosphorylation (OXPHOS) in mature adipocytes, with increasing reliance on fatty acid oxidation and the TCA cycle. This transition supports enhanced fatty acid synthesis, lipid droplet accumulation, and establishment of long-term energy storage [129–131].

Mitochondria are central metabolic hubs during adipogenesis and play a critical role in supporting the dynamic metabolic demands associated with adipocyte differentiation [76]. In mouse 3T3-L1 preadipocytes, adipogenic differentiation is accompanied by extensive mitochondrial remodeling, including changes in mitochondrial number, morphology, and metabolic capacity [77]. The changes are presented in Fig. 4. Functionally, mitochondria support de novo triglyceride synthesis by providing acetyl-CoA, generated in part through pyruvate carboxylase–dependent anaplerotic flux, underscoring their indispensable role in lipid accumulation during adipogenesis [78].

Adipocyte differentiation requires a coordinated increase in mitochondrial enzyme expression, inner membrane expansion, and overall oxidative capacity [78]. At the structural level, differentiation is associated with the upregulation of components of the mitochondrial contact site and cristae organizing system (MICOS; mitochondrial contact site and cristae organizing system), a multi-protein complex that governs cristae junction formation and inner membrane architecture. Proper cristae organization is essential for maintaining a large membrane surface area to accommodate oxidative phosphorylation complexes (Complexes II–IV) and ATP synthase, thereby supporting the elevated bioenergetic and biosynthetic demands of differentiating adipocytes [79].

Pharmacological activation of adipogenesis further highlights the link between mitochondrial dynamics and adipocyte plasticity. Thiazolidinediones (TZDs), potent PPARγ agonists, robustly induce adipocyte differentiation in vitro, although their precise downstream mechanisms remain incompletely defined. Notably, rosiglitazone treatment alters both mitochondrial abundance and morphology, suggesting that mitochondrial biogenesis and structural remodeling are integral components of PPARγ-driven adipogenic programs [80].

Given the close association between mitochondrial structure and cell fate determination, metabolic flexibility, and adaptation to environmental stress, alterations in mitochondrial homeostasis alone are sufficient to drive adipocyte identity remodeling [81]. For instance, mitochondrial stress can induce a “whitening” transition in brown adipocytes. In brown adipose cells, selective loss of mitochondrial proteases leads to a marked increase in lipid droplet number accompanied by lipid droplet enlargement, resulting in phenotypic features characteristic of white adipocytes. Ultrastructural analyses further reveal that this process is associated with an increased number of mitochondria exhibiting abnormal morphology, including enlarged size, reduced electron density, and diminished cristae structure, indicative of profound mitochondrial structural and functional remodeling [82]. Collectively, these observations highlight that mitochondria are not merely organelles for energy metabolism but are key determinants in maintaining adipocyte identity and cellular state stability. Thus, existing studies establish mitochondria as active regulators of adipocyte differentiation and metabolic identity, while simultaneously highlighting a critical unmet need to define how mitochondrial structure and function are reorganized during adipocyte dedifferentiation. Elucidating these mechanisms may provide important insights into how adipocytes transition from a lipid-storage phenotype to a metabolically plastic, stress-responsive state under pathological conditions such as cancer or metabolic disease.

Overall, comparative analysis of metabolic changes during adipocyte differentiation and dedifferentiation not only deepens our understanding of cell identity across distinct differentiation states, but also provides a new conceptual framework for identifying and regulating the dedifferentiation process. During differentiation, adipocytes undergo well-defined metabolic trajectory shifts. For example, at the preadipocyte stage, metabolism is primarily geared toward supporting proliferation and membrane biogenesis, with a preference for the synthesis of long-chain saturated fatty acids. In contrast, mature adipocytes adopt a storage-centered metabolic program, in which the fatty acid synthesis profile progressively shifts toward unsaturated fatty acids to accommodate lipid droplet expansion and long-term energy storage. In parallel with these metabolic changes, nuclear morphology, chromatin organization, and transcriptional activity are systematically remodeled, collectively constituting key phenotypic features that distinguish adipocyte differentiation states.

Systematic characterization of metabolic and structural features across differentiation stages provides multidimensional criteria for defining adipocyte identity, an approach that can be readily extended to the study of dedifferentiation. By comparing the metabolic features of dedifferentiated adipocytes with those of canonical differentiation stages, it becomes possible to assess whether these cells have truly departed from a mature adipocyte state and transitioned toward a more plastic cellular identity.

Moreover, contrasting the distinct metabolic reprogramming events that occur during differentiation and dedifferentiation offers important insights into the mechanisms governing dedifferentiation. Whereas differentiation reflects the directional establishment of a defined metabolic program, dedifferentiation is more prominently characterized by adaptive metabolic remodeling in response to energetic stress and microenvironmental cues. Elucidating how these metabolic axes are rewired will not only advance our understanding of adipocyte plasticity but also provide a rationale for future strategies aimed at modulating adipocyte dedifferentiation, transdifferentiation, or functional remodeling through metabolic intervention.

### Genetics and epigenetics in adipocyte differentiation and dedifferentiation

Genetic and epigenetic mechanisms jointly regulate adipocyte differentiation and dedifferentiation by linking gene expression, protein abundance, and environmental factors [83, 84]. Epigenetic modifications such as DNA methylation, histone acetylation/methylation, and chromatin remodeling play key roles in adipogenesis by regulating transcriptional reprogramming [85].

### Genetic

Transcription factors like C/EBPα and PPARγ drive adipogenesis by activating key genes [86]. Pre-adipocytes exit the cell cycle, express adipocyte-specific genes, accumulate lipids, and ultimately acquire mature adipocyte morphology [87]. PPARγ maintains adipocyte identity; its inhibition in 3T3-L1 adipocytes induces dedifferentiation, lipid loss, and downregulation of adipocyte markers [88]. In vivo, Pparg knockout triggers adipocyte loss, followed by regeneration [89]. C/EBPβ deficiency reduces adipose tissue mass, confirming its role in adipogenesis [90].

The regulatory mechanisms of adipocyte dedifferentiation are not yet fully understood, but existing research suggests that several signaling pathways, including Wnt/β-catenin, TGF-β, Notch, and Hippo, regulate adipocyte dedifferentiation. Wnt3a and TGF-β suppress adipocyte genes, reverting cells to a progenitor-like state [26].

### Histone modifications and gene expression

**Fig. 5:**
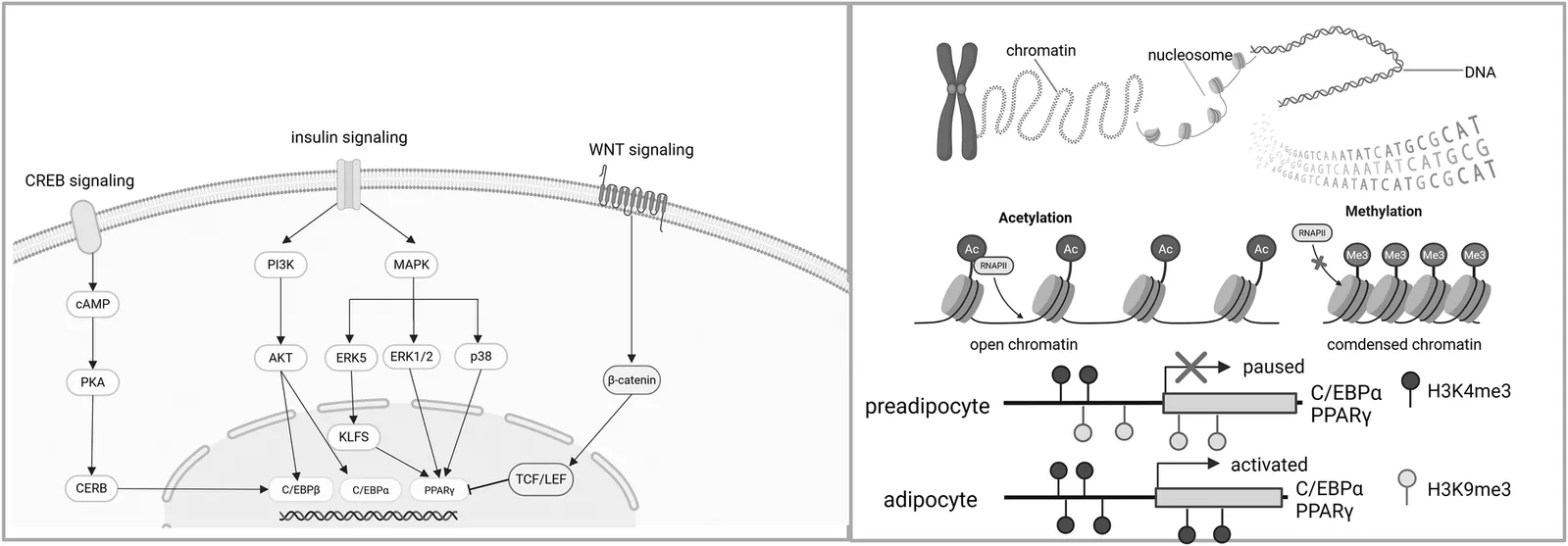
Epigenetic regulation of chromatin states during adipocyte differentiation. Histones wrap DNA to form nucleosomes, which are further compacted into chromatin and ultimately chromosomes. Methylation induces chromatin condensation and suppresses gene expression, whereas acetylation relaxes chromatin structure, increasing DNA accessibility and promoting transcription. During adipocyte differentiation, classical bivalent domains pair H3K4me3 with H3K9me3 to maintain key adipogenic regulators (such as *Cebpa* and *Pparg*) at a low expression state. These bivalent chromatin marks preserve a reversible repressive state by preventing irreversible promoter silencing, thereby keeping the genes poised for activation upon differentiation cues.

Histone acetylation is positively correlated with gene expression, and various histone modifications can collaboratively regulate transcription by marking the same histone residues. In some cases, the same chromatin region can simultaneously carry both repressive and active histone modifications [91]. For instance, antagonistic histone modifications such as H3K4me3 (active) and H3K27me3 (repressive) are crucial in early development. These bivalent marks maintain lineage-specific genes in a poised but repressed transcriptional state in stem and progenitor cells [92, 93]. In pre-adipocytes, bivalent marks such as H3K4me3 and H3K9me3 are found at the C/EBPα and PPARγ gene loci, preparing these genes for active expression and triggering the initiation of adipocyte differentiation [94, 95], as shown in Fig. 5.

Beyond differentiation, epigenetic regulation also plays a crucial role in adipocyte dedifferentiation. For instance, Keung et al. demonstrated that suppression of the tumor suppressor gene KLF6 in liposarcoma cells led to increased H3K9me3 levels. This, in turn, repressed the expression of PPARγ and CEBPα, thereby promoting dedifferentiation [96]. These findings highlight the potential of epigenetic mechanisms to regulate adipocyte fate. Moreover, alterations in the cellular microenvironment, such as changes in culture conditions, may induce dedifferentiation through epigenetic remodeling.

### New mechanistic insights from advanced technology

#### Traditional and emerging technologies for adipocyte dedifferentiation

Adipocyte dedifferentiation is a complex biological process. Traditional techniques such as BODIPY and Oil Red O staining have been widely used to visualize intracellular lipid droplets and track changes during dedifferentiation., Quantitative PCR (qPCR) is commonly used to measure the expression of genes associated with adipocyte differentiation (e.g., PPARγ, FABP4, ADFP), mesenchymal stem cells (e.g., Vimentin, CD44, Sox9), and lipid metabolism (e.g., FASN, HSL, LPL) [97]. Western blotting enables the quantification of proteins involved in lipid metabolism and signaling pathways, such as FASN, HSL, LPL, and those in the Wnt/β-catenin, MAPK, and TGF-β pathways [98].

Despite their importance, these methods are invasive and provide static snapshots. Staining and molecular extraction disrupt cells and cannot reflect dynamic, real-time changes during dedifferentiation. This limitation underscores the need for emerging technologies that offer higher resolution and temporal precision.

#### Single-cell transcriptomics

The definition of the various types of cell fate changes, including cell expansion, differentiation, transdifferentiation, dedifferentiation, reprogramming, and state transitions, represents a complex and evolving field of research known as cell lineage tracing [99]. Single-cell transcriptomics has revolutionized research by enabling the study of gene expression at the single-cell level, revealing cellular behaviors and their mechanisms [100]. Single-cell sequencing reveals that even under similar conditions, cells exhibit significant differences in differentiation, metabolism, and epigenetic regulation. For example, epidermal white adipocytes become “dedifferentiated-like cells” during local infections, hair cycling, or wound healing [25, 101, 102]. By analyzing the transcriptomes of white adipocytes from different regions of the body, it was found that adipocyte progenitors (FAPs) from different fat depots have varying adipogenic potentials [29]. In visceral white adipose tissue, FAPs exhibit robust adipogenic differentiation potential, while FAPs in gonadal white adipose tissue lack adipogenic capability in both in vivo and in vitro transplantation experiments [103, 104].

Another important application of single-cell transcriptomics is the reconstruction of cellular dynamics. In this approach, individual cells are ordered along a time trajectory (or pseudo-time) based on the similarity of their gene expression profiles, helping to understand different stages of in vivo adipogenesis [105]. A trajectory of adipogenesis was mapped, including early pre-adipocytes, late pre-adipocytes, transitional cells, and mature adipocytes. A unique subgroup present in late pre-adipocytes was found to be highly sensitive to differentiation stimuli. Projection of differential gene expression on the pseudo-time scale revealed the sequential expression of gene programs during in vivo adipogenesis, including the temporal expression of transcriptional regulators that may drive adipocyte gene activation [106]. Integrating this with single-cell epigenomic studies will help deepen the understanding of the transcriptional mechanisms driving cell transitions, including adipogenesis.

Furthermore, single-cell sequencing can be used for lineage tracing, and our previous ideas about the gradual differentiation of adipocytes have evolved. Adipocyte progenitors and pre-adipocytes follow distinct differentiation pathways: adipocyte progenitors have stronger potential to differentiate into mature adipocytes, while pre-adipocytes, maintaining characteristics of immature adipocytes, may possess greater plasticity or adaptability to environmental changes, enabling them to respond rapidly to injury signals [107]. This feature of pre-adipocytes holds potential for them to replace adipose stem cells in cases of localized fat loss, fibrosis, or wound healing.

#### Proteomics

Recent advancements in transcriptomics have significantly enhanced our understanding of adipocyte heterogeneity and the transcriptional networks underlying adipogenesis [106], mRNA levels do not directly correlate with protein abundance, and post-translational modifications (PTMs) introduce additional layers of regulation beyond transcription [108, 109]. PTMs are crucial for adipocytes to adapt to their environment and function properly [110, 111]. Post-translational modifications (PTMs) provide a rapid and reversible mechanism for regulating adipocyte differentiation and dedifferentiation by modulating the activity, stability, and subcellular localization of key transcription factors and metabolic enzymes. Among these, phosphorylation is the most extensively studied. For example, phosphorylation of PPARγ by MAPK at Ser112 reduces its transcriptional activity and suppresses adipocyte differentiation, whereas dephosphorylation enhances adipogenic gene expression. Similarly, insulin-AKT signaling promotes adipogenesis in part through phosphorylation-dependent activation of downstream metabolic and transcriptional regulators. Acetylation and deacetylation also play critical roles in adipocyte fate control. Acetylation of PPARγ and C/EBPα enhances their transcriptional activity, while deacetylation by SIRT1 represses adipogenic gene expression and promotes lipolysis, a mechanism implicated in adipocyte dedifferentiation and metabolic stress responses [112]. In line with this, SIRT1 activation has been shown to destabilize the mature adipocyte phenotype and facilitate a more plastic, less differentiated state. Proteomics enables deeper insights into adipocyte function and metabolic regulation, High-throughput proteomics now allows for more precise protein profiling in adipose tissue.

Furthermore, compared to transcriptomics, proteomics captures protein localization, dynamics, and interactions, overcoming traditional technique limitations. Most cellular biological processes involve changes in the subcellular localization of proteins, and mislocalization is linked to diseases like neurodegeneration, cancer, and metabolic disorders [113–115]. Capturing protein spatial distribution and dynamics is essential for understanding cell biology. Spatial proteomics reveals dynamic protein relocalization, including nuclear-cytoplasmic shuttling, mitochondrial shifts during apoptosis, and receptor endocytosis [116].

#### Multi-omics

Adipocyte identity transition is a complex, dynamic process that spans transcriptional regulation, proteostasis, metabolic reprogramming, and organelle remodeling. Single-omics approaches are insufficient to comprehensively capture these multilayered dynamics. Integrated multi-omics analyses, which simultaneously interrogate the transcriptome, epigenome, proteome, and metabolome, have therefore emerged as essential tools for elucidating the molecular basis of adipocyte differentiation, dedifferentiation, and transdifferentiation, particularly the processes of identity loss, reprogramming, and stabilization [117].

In studies of adipocyte differentiation, the combined application of transcriptomic and epigenomic approaches—such as single-cell RNA sequencing (scRNA-seq) and single-cell ATAC sequencing (scATAC-seq)—has successfully reconstructed the continuous trajectory from preadipocytes to mature adipocytes and identified stage-specific regulatory networks driven by key transcription factors, including PPARγ and C/EBPα [118, 119]. However, proteomic analyses have further revealed that the establishment of adipocyte identity is not solely dependent on transcriptional activation. Instead, it is accompanied by profound changes in protein abundance, subcellular localization, and organelle-associated protein networks, particularly involving lipid droplets, mitochondria, and cytoskeletal components [75, 120]. Notably, these protein-level alterations are often not fully reflected at the transcriptional level, highlighting the indispensable role of post-transcriptional and proteome-level regulation in determining adipocyte fate.

### Challenges and perspectives

Obesity is driven by adipocyte hypertrophy and hyperplasia. During dedifferentiation, lipid droplets are consumed, releasing fatty acids for oxidation and reducing cellular lipid burden, thereby lowering whole-body fat. Unlike brown adipose tissue, which relies on UCP1-mediated thermogenesis and external stimuli (cold, β3-agonists) with lineage restrictions, dedifferentiation offers greater plasticity. Mature white adipocytes can revert to progenitor-like states under stress (energy deprivation, mechanical cues, signaling activation), accompanied by lipid clearance, metabolic reprogramming, and stemness acquisition. Dedifferentiated cells also improve insulin resistance by enhancing lipolysis, autophagy, and anti-inflammatory cytokine secretion.

However, the phenotype and trajectory of dedifferentiation remain undefined, and no stable in vivo induction method exists. In vitro, TGF-β induces dedifferentiation via Hippo-YAP/TAZ-TEAD signaling, but excessive activation causes fibrosis [44]. Dedifferentiated adipocytes may also promote tumor progression, contributing to liposarcoma, breast, and ovarian cancer through extracellular matrix remodeling [17].

The biological uncertainties and potential safety risks in adipocyte dedifferentiation substantially hinder clinical translation. In practice, application is further constrained by the high manufacturing and quality-control costs associated with cell isolation, expansion, and phenotypic validation, as well as regulatory uncertainties regarding cell identity, stability, and long-term safety, and ethical concerns related to donor variability and clinical-grade cell manipulation. These challenges collectively underscore the need for standardized induction protocols, molecular criteria to define dedifferentiated adipocyte states, and unified clinical frameworks to ensure reproducibility, safety, and regulatory compliance.

## Conclusion

Adipocyte dedifferentiation, as a unique process of cell fate transformation, represents an important source of pluripotent stem cells and provides new perspectives for both basic research and clinical applications, DFAT cells have been explored as a potential cell source for bone, cartilage, muscle, and adipose tissue regeneration, as well as for wound healing and tissue reconstruction, owing to their accessibility, autologous origin, and multilineage differentiation capacity. Given the limited research on adipocyte dedifferentiation in the past, this review aims to clarify the dedifferentiated state of adipocytes and the metabolic pathway adjustments during this process. By examining intracellular substance changes, organelle remodeling, and metabolic regulation at different stages of dedifferentiation, we hope to provide key insights into this cellular transformation. The combination of single-cell omics techniques with high-throughput analysis methods offers new directions for the identification and application of dedifferentiated adipocytes, which enables systematic comparisons of dedifferentiated adipocytes generated under different induction conditions, from distinct donors, and across independent production batches, thereby providing a molecular basis for consistency assessment and quality. In conclusion, these findings, in conjunction with emerging technologies, offer many opportunities to better understand adipocyte fate conversion and its potential clinical therapeutic strategies.

## Data Availability

Data sharing is not applicable to this article as no datasets were generated or analyzed during the current study.
